# Midkine promotes glioblastoma progression via PI3K-Akt signaling

**DOI:** 10.1186/s12935-021-02212-3

**Published:** 2021-09-23

**Authors:** Beiquan Hu, Chao Qin, Li Li, Lei Wei, Xianlun Mo, Huasheng Fan, Yuanhua Lei, Feng Wei, Donghua Zou

**Affiliations:** 1 Department of Neurosurgery, The Fifth Affiliated Hospital of Guangxi Medical University, Nanning, 530022 Guangxi People’s Republic of China; 2 Intensive Care Unit, The People’s Hospital of Cangwu, Wuzhou, 543100 Guangxi People’s Republic of China; 3 Department of Neurology, The Fifth Affiliated Hospital of Guangxi Medical University, Nanning, 530022 Guangxi People’s Republic of China; 4 Department of Neurology, The First People’s Hospital of Nanning, Nanning, 530022 Guangxi People’s Republic of China

**Keywords:** Glioblastoma, GBM, MDK, Midkine, PI3K-Akt signaling, Differentially expressed genes, Migration, Invasion

## Abstract

**Background:**

Our previous bioinformatics-based study found that midkine (MDK) was associated with poor prognosis of glioblastoma (GBM). However, the mechanism of MDK in GBM remains elusive.

**Methods:**

A public GBM-related dataset and GBM tissues from our center were used validate the aberrant expression of MDK in GBM at the RNA and protein levels. The relationship between MDK expression and survival of GBM patients was also explored through survival analysis. Subsequently, we identified MDK-related GBM-specific genes using differential expression analysis. Functional enrichment analyses were performed to reveal their potential biological functions. CCK-8, 5-ethynyl-2′-deoxyuridine, and Matrigel-transwell assays were performed in GBM cell lines in which MDK was knocked out or overexpressed in order assess the effects of MDK on proliferation, migration, and invasion of GBM cells. Western blotting was performed to detect candidate proteins.

**Results:**

Our study showed MDK is a promising diagnostic and prognostic biomarker for GBM because it is highly expressed in the disease and it is associated with poor prognosis. MDK is involved in various cancer-related pathways, such as PI3K-Akt signaling, the cell cycle, and VEGF signaling. A comprehensive transcriptional regulatory network was constructed to show the potential pathways through which MDK may be involved in GBM. In vitro, Overexpression of MDK augmented proliferation, migration, and invasion of GBM cell lines, whereas suppression of MDK led to the opposite effects. Furthermore, our study confirmed that MDK promotes the progression of GBM by activating the PI3K-Akt signaling pathway.

**Conclusions:**

Our present study proposes that MDK promotes GBM by activating the PI3K-Akt signaling pathway, and it describes a potential regulatory network involved.

**Supplementary Information:**

The online version contains supplementary material available at 10.1186/s12935-021-02212-3.

## Background

Glioblastoma (GBM) is the most common malignant tumor in the brain and spinal cord [1]. Even though great progress has been made in the treatment of GBM, its mortality remains near 95% [2]. Studies have shown that high invasiveness is one of the most important factors contributing to the low survival rate of GBM patients [3]. Because of the invasive nature of GBM, surgical resection is unlikely to capture every cancer cell. Even if the tumor mass is removed, risk of GBM recurrence remains high. While a patient may undergo subsequent surgical resections, this therapeutic strategy only prolongs survival and is not curative. Understanding the mechanisms that contribute to GBM’s invasiveness may provide new insights for targeting the metastasized cells, thereby transforming the treatment of GBM.

Several studies have shown that gene differential expression or mutation is associated with the risk of GBM which include Genetic variants in m6A modification core genes [4], polymorphism of KARS gene [5]. In our previous bioinformatics-based study, we identified several transcription factors as well as growth factors and cytokines (GFCK) that are differentially expressed in GBM and that are related to the prognosis of GBM patients [6, 7]. One of these proteins is midkine (MDK). MDK is a heparin-binding growth factor that promotes cell proliferation [8], migration [9], and angiogenesis [10] during tumorigenesis. MDK can interact with notch 2, which promotes interaction between HES1 and STAT3 as well as the epithelial-mesenchymal transition (EMT) [11]. The EMT is a process by which the cell becomes more migratory and is a key step in cancer metastasis. Similarly, the gene encoding midkine can be alternately spliced to yield transcripts of multiple isoforms [12], and these abnormal transcription factors usually lead to disease. We can explore the abnormal expression of transcription factors in GBM, and then probe their role in the development of GBM.

MDK has been shown to be overexpressed in several tumors, including non-small cell lung cancer, thyroid cancer, and GBM [13, 14]. The overexpression of MDK may lead to multidrug resistance in gastric cancer [15]. The frequent upregulation of MDK across tumor types may reflect that the gene’s promoter contains a hypoxia response element [16]. The mechanism of MDK in GBM remains elusive, highlighting the need for further studies.

In order to clarify the role of MDK in GBM, we verified in the present study the increased expression of MDK RNA and protein in tumors, and we found that high MDK expression is associated with poor prognosis of GBM patients. We constructed a comprehensive transcriptional regulatory network showing potential pathways through which MDK is involved in GBM, and we identified four transcription factors (REST, TCF12, SP1, and XRN2) that may be direct interactors with MDK. Cell proliferation, migration, and invasion were investigated in GBM cell lines over- and under-expressing MDK. The results of our study suggest that MDK promotes GBM, and our data lead us to propose potential mechanisms.

## Materials and methods

### Patients

A total of 94 GBM patients (45 males and 49 females) were recruited between January 2005 and December 2013 from the Fifth Affiliated Hospital of Guangxi Medical University. GBM patients were classified according to World Health Organization (WHO) criteria, and their diagnosis was confirmed in the present study based on histology analysis by two pathologists working independently. Cancerous tissue and normal brain tissue samples were collected from GBM patients. This study was approved by the Ethics Committee of the Fifth Affiliated Hospital of Guangxi Medical University. The requirement for informed consent was waived for the present study because at the time of treatment, patients gave written consent for their anonymized medical data to be analyzed and published for research purposes.

### Public data collection and processing

The GSE50161 [17] dataset was downloaded from the Gene Expression Omnibus (GEO) database [18] and included gene expression profiles of 34 GBM samples and 13 normal brain samples, based on the GPL570 platform. Normal brain samples were obtained from pediatric epilepsy patients (median age 13 years) at the time of surgical intervention. This dataset was used in the analysis of differentially expressed genes (DEGs). The normalizeBetweenArrays function in the limma package [19] in R was used to normalize the gene expression profiles between individuals. If a gene was measured by multiple probes, the average expression value of these probes was used. The glioma datasets mRNAseq_693 (last update: November 28, 2019) containing mRNA sequencing (mRNA-seq) data and anonymized clinical information were downloaded from the Chinese Glioma Genome Atlas [20].

### Analysis of DEGs and receiver operating characteristic (ROC) curves

We used the limma package [19] in R to identify the DEGs between GBM samples and healthy brain tissue in GSE50161, and the DEGs between GBM samples showing high or low MDK levels, with the cut-off defined as the median level. Differences associated with P < 0.05 (adjusted by the false discovery rate) were considered significant. Genes that were up- or downregulated in both pairwise comparisons were considered to be MDK-related, GBM-specific genes. The GEPIA web tool [21] was used to validate the aberrant expression of MDK using data from The Cancer Genome Atlas (TCGA) [22, 23] and Genotype-Tissue Expression (GTEx) database [24]. Finally, ROC curves were analyzed using the pROC package [25] to determine whether MDK had the potential to be a diagnostic marker for GBM.

### Immunohistochemistry

Two pathologists who were blind to the clinicopathological information independently evaluated the GBM and health brain tissue sections of each patient. The percentage of MDK-positive cells were scored (0 = 0% of tumor cells were positive, 1 = 0–10%, 2 = 11–50%, 3 = 51–75%, 4 = 75–100%), and MDK staining intensity was scored (0 = negative, 1 = weak, 2 = medium, 3 = strong). These two scores were multiplied to get a total score from 0 to 12. According to protein level expression of MDK, the patients were divided into MDK-low patients (total score < 4) and MDK-high patients (total score ≥ 4).

### Survival analysis

Survival was calculated starting from the date of surgery to date of death or last follow-up. Follow-up data lasting a mean of 14.74 ± 13.13 months (range, 0.03–59 months) were available for 43 patients, and all other patients were excluded from the survival analyses. Survival analysis was also conducted in a GBM dataset (mRNAseq_693) from the Chinese Glioma Genome Atlas database [20] to explore the relationship between MDK expression and survival of GBM patients. Survival curves for different levels of MDK in GBM patients were plotted using the Kaplan–Meier method [26] and compared using the log-rank test. Differences associated with P < 0.05 were considered significant.

### Functional enrichment analysis

In order to identify potential biological processes and pathways of the MDK-related, GBM-specific genes, Gene Ontology (GO) and Kyoto Encyclopedia of Genes and Genomes (KEGG) enrichment analysis was performed using the clusterProfiler [27] package in R. To determine whether the candidate signaling pathway was activated in MDK-high patients, we applied the gene set variation analysis (GSVA) to score these genes using the GSVA package [28].

### Construction of the regulatory network of MDK in promoting GBM

The transcription factors (TFs) that interact with MDK were obtained from the RNAInter database [29]. The Pearson expression correlation of candidate TFs and MDK were explored, and P < 0.05 and R > 0.7 were applied as thresholds. Furthermore, we downloaded the structures of MDK and its interacting TFs from the Protein Data Bank (PDB) database [30, 31]. The relationship of MDK and its TFs was explored using hex software (version 8.0.0) [32], and the results were visualized using PyMOL software [33]. To construct the MDK-related regulatory network, genes interacting with the candidate TFs were also included. The network was visualized using Cytoscape software [34].

### Cell lines

The human GBM cell lines U87-MG, U251-MG, A172, and T98G were purchased from the American Type Culture Collection (ATCC). The cell lines were cultured in Dulbecco’s Modified Eagle Medium (DMEM; Invitrogen, Carlsbad, CA, USA) supplemented with 10% fetal bovine serum (FBS, Invitrogen) at 37 °C and humidified atmosphere with 5% carbon dioxide. The medium was changed every 2 days and cells were split when they reached 80–90% confluence; for splitting, cells were collected by adding 0.25% trypsin for 5 min. Cell lines were discarded after passage 3. The protein and gene expression of MDK in GBM cell lines was quantified by Western blot and quantitative real-time polymerase chain reaction (qPCR).

### Lentiviral transfection for MDK knockdown and overexpression

To maintain physiologically relevant levels of MDK while varying its expression, we knocked down MDK in high expression cell lines and overexpressed MDK in low expression cell lines. Lentiviral vectors encoding small hairpin RNA (shRNA) targeting human MDK were constructed by Hanyin Co. (Shanghai, China), and these vectors also encoded green fluorescent protein (GFP). Lentiviral vectors encoding GFP alone were also prepared as a negative control (NC). In order to obtain stable MDK knockdown cell lines (MDK-KD) and stable NC cell lines, we inoculated U87-MG and T98G cells in a 6-well plate, and then infected them on the following day at a multiplicity of infection of 1 in the presence of 8 μg/ml Polybrene. About 72 h after infection, GFP expression was observed under fluorescence microscopy, and the medium was changed to selection medium containing puromycin (4 μg/ml) (catalog no. MS0011-25MG, Shanghai MaoKang Biotechnology). The cells were cultured for at least 14 days in selection medium, then the puromycin dose was lowered to 2 μg/ml for amplification for 7–9 days. Subsequent experiments were performed in DMEM without puromycin. The efficiency of anti-MDK shRNA was verified by qPCR and Western blot.

We constructed a lentiviral vector encoding Flag-tagged MDK based on the pMSCV-IRES-GFP vector (Hanyin Co.), as well as the corresponding NC lentiviral vector encoding only GFP. The GBM cell lines A172 and U251-MG were infected with the recombinant lentivirus at a multiplicity of infection of 1, resulting in MDK overexpression (MDK-OE) cells or NC cells. MDK expression was verified by fluorescence microscopy, qPCR, and Western blot.

### qPCR

Total RNA from U87-MG, U251-MG, A172, and T98G cells was isolated using TRIzol (catalog no. 15596-018, Life Technologies). For qPCR analysis, 500 ng of total RNA was reverse-transcribed into cDNA using a miScript II RT Kit (Qiagen, 218161), which was amplified by PCR involving 40 cycles of 5 s at 95 °C and 30 s at 60 °C. Differences in gene expression were determined by the 2^−ΔΔCT^ method against an endogenous control used for calibration [35]. The primer sequences were as follows: human MDK-F, CGCGGTCGCCAAAAAGAAAG; human MDK-R, ACTTGCAGTCGGCTCCAAAC; human GAPDH-F, GTCTCCTCTGACTTCAACAGCG; human GAPDH-R, ACCACCCTGTTGCTGTAGCCAA.

### Western blotting

We explored the expression of MDK, phosphorylated Akt (p-Akt), Akt, phosphorylated ERK (p-ERK), ERK, phosphorylated PI3K (p-PI3K), and PI3K in GBM cell lines by western blot as described previously [36–38]. Primary antibodies included mouse antibody against actin (Millipore, MA, USA; 1:10,000) and rabbit antibodies (Proteintech, IL, USA; 1:1000) against human MDK (14958-1-AP), p-Akt, Akt, p-ERK, ERK, p-PI3K or PI3K.

### Cell proliferation assays

Cell Counting Kit-8 (CCK-8) assay (Beyotime, Shanghai, China) was used to detect cell proliferation over 5 days. After the cells (n = 2000) were seeded into 96-well plates, CCK-8 solution (10 μl) was added to each well at different time points, and plates were cultured at 37 °C for 2 h. The absorbance at 450 nm was measured using a microtiter plate reader. The experiment was repeated three times to ensure reproducibility. We performed a commercial proliferation assay based on 5-ethynyl-2′-deoxyuridine (EdU) according to the manufacturer's instructions (Invitrogen, Carlsbad, California, USA).

### Transwell migration assay

Cells (U87-MG, U251-MG, A172, and T98G) were seeded into the top chamber of 24-well Transwell plates with 8-µm inserts (catalog no. 3422, Corning). Conditioned medium from the same GBM cells was added to the bottom chamber. After 12-h incubation, the cells that migrated through the membrane were fixed in methanol and stained with 1% crystal violet (Beyotime Biotechnology, C0121). The cells were counted in four random fields.

### Matrigel-transwell invasion assays

Diluted Matrigel solution was added to serum-free DMEM in the upper chamber of 24-well Transwell plates (catalog no. 3422, Corning), then cells were plated in the upper chamber. The inserts were then placed in the bottom chamber containing DMEM supplemented with 10% FBS as a chemoattractant. After 24 h, the top of the insert was wiped with a sterile cotton swab to remove the unmigrated cells. The remaining cells were stained with 0.1% crystal violet for 1 h. The cells were examined, counted, and imaged under a microscope. The numbers of cells in five random fields of each chamber were counted and averaged.

### Co-immunoprecipitation (CO-IP) assay

CO-IP enables the purification of proteins based on the formation of antigen/antibody complexes. Thus, proteins can be isolated and purified from the rest of the sample to study the direct interaction between the protein and the specific binding partner.

MDK overexpressing A172 cells were collected and lysised, immunoprecipitation was performed by adding target antibodies of MDK or control antibody, use immunoglobulin (IgG) as a guideline. Then adding the Protein-G Sepharose (P-GS) beads (Sigma, USA) to precipitate the protein complex. P-GS beads (Sigma, USA) were washed three times with Protein Extraction Buffer prior to mixing with the antibody. The concentration of P-GS beads used for immunoprecipitation was 10% (v/v). After mix purified antibody against MDK target protein or control antibody with 100 µl of P-GS bead solution and incubate for 15 min at room temperature. Incubate on a rocking chair for 15 min. The prepared antibody P-GS is added to 1 ml of the extract and incubated for 2 h at 4 °C with shaking. Centrifuge the immunoprecipitate at 130×*g* for 3 min and discard the supernatant. Wash the pellet by resuspension and centrifugation three times with extraction buffer. The bound protein is released from the protein-G antibody by adding 2 × SDS gel loading buffer and boiling for 4 min. After centrifugation, an equal volume of each sample was fractionated by SDS-PAGE.

### Statistical analyses

All statistical analyses were performed using R [39]. We analyzed the expression levels of genes using unpaired t-tests. Survival curves for MDK were plotted using the Kaplan–Meier method and compared using the log-rank test. All samples were analyzed in triplicate in all experiments. Differences associated with a two-tailed P < 0.05 were considered statistically significant.

## Results

### MDK expression in GBM tissues

**Fig. 1:**
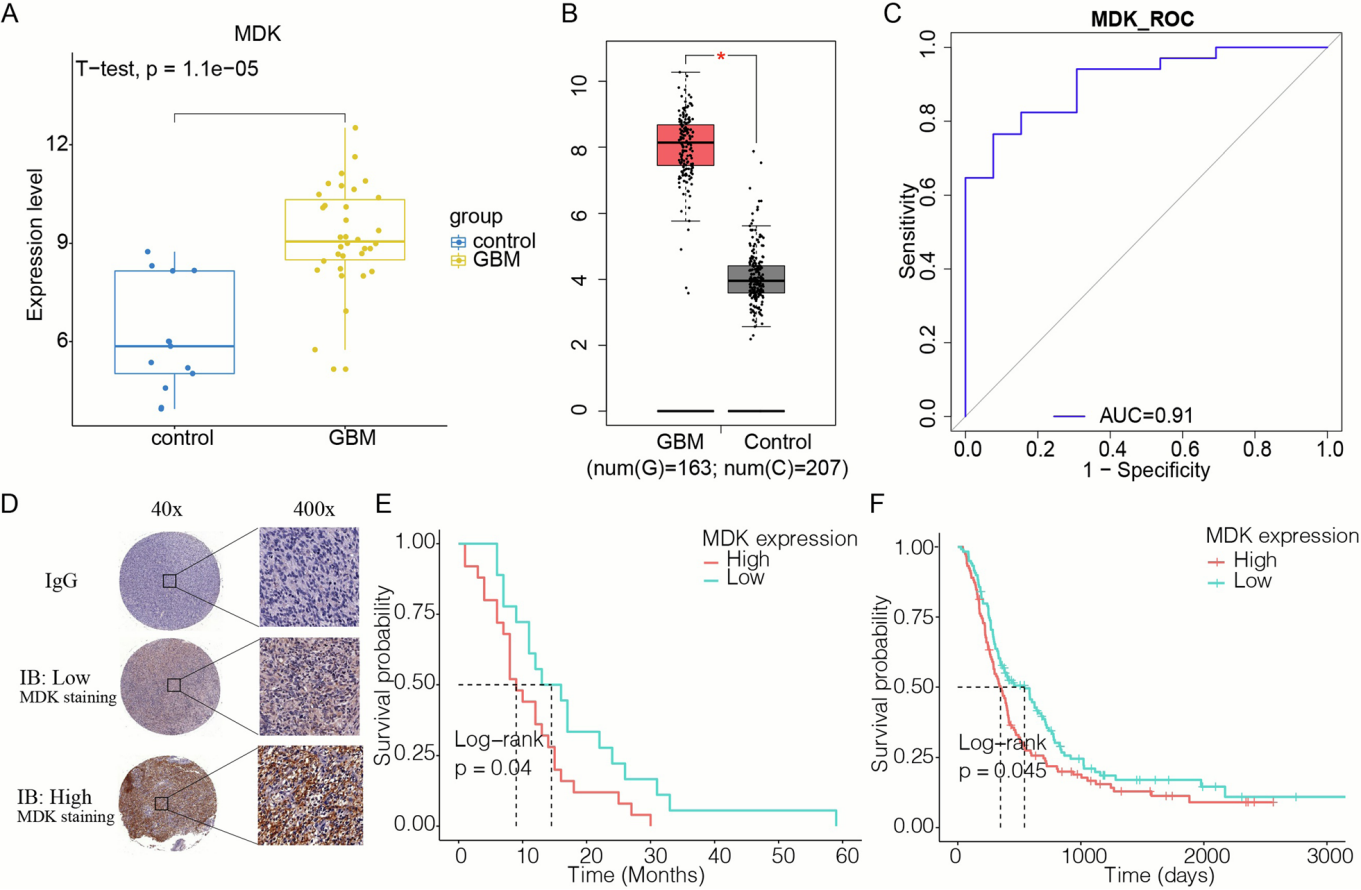
Expression of MDK in GBM as a diagnostic and prognostic biomarker. **A** The expression of MDK in healthy brain is lower than in GBM samples in GSE50161. **B** The expression of MDK in normal control samples (n = 207) is lower than in GBM samples (n = 163) in the TCGA and GTEx datasets. * p < 0.05. **C** The receiver operating characteristic (ROC) curve of MDK as a biomarker of GBM in GSE50161. **D** Representative images of immunohistochemical staining in tumor sections of patients with GBM. MDK showed high and low expression in GBM tissues from different patients. Immunoglobulin (IgG) was used as a negative control. **E** Kaplan–Meier curves of overall survival for 43 GBM patients, divided by MDK expression. **F** Kaplan–Meier curves of overall survival for GBM patients in the CGGA database, stratified by MDK expression. MDK: midkine; GBM: glioblastoma; TCGA: The Cancer Genome Atlas; GTEx: Genotype‑Tissue Expression; CGGA: Chinese Glioma Genome Atlas; IB: Immunoblotting

MDK RNA expression was higher in GBM brain tissue than in healthy controls in three independent sets of patients from the CGGA, TCGA and GTEx databases (Fig. 1A, B). ROC curve analysis of the RNAseq data demonstrated that MDK has potential to be a valuable diagnostic biomarker of GBM: it gave an area under the ROC curve (AUC) of 0.91 (Fig. 1C). The level of MDK protein in tumors varied with the patient (Fig. 1D). When we divided patients into subgroups based on the level of MDK gene expression, we found that MDK-positive GBM patients had worse prognoses than those with MDK-negative GBM (P = 0.04) (Fig. 1E). This correlation was also present in CGGA datasets (P = 0.045, Fig. 1F).

### MDK-related, GBM-specific genes and their functional pathways

**Fig. 2:**
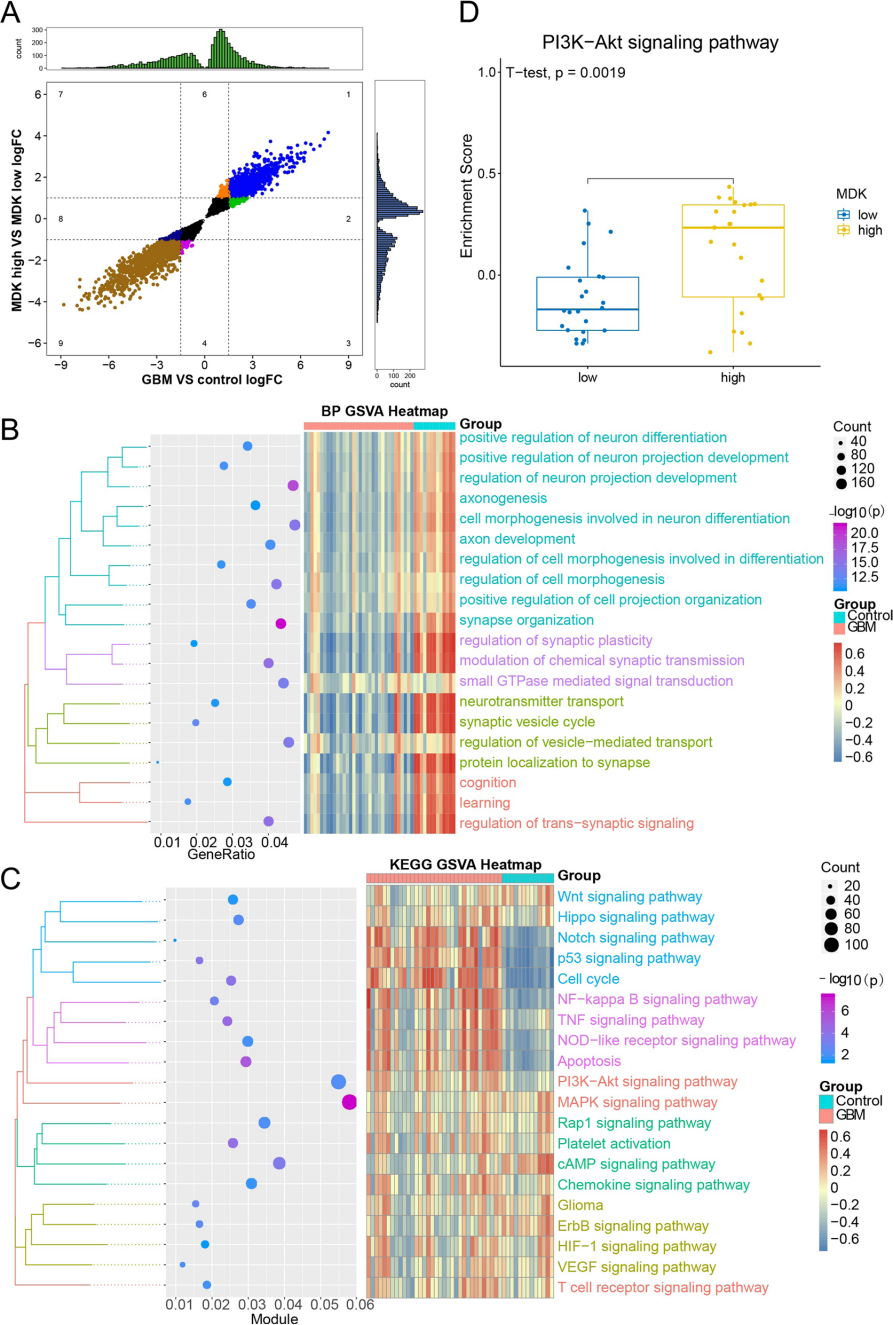
Gene Ontology (GO) and Kyoto Encyclopedia of Genes and Genomes (KEGG) enrichment analysis of MDK-related, GBM-specific genes. **A** Quadrant plot of identified MDK-related and/or GBM-specific genes. The x- and y-axes show the expression values of two groups of different genes, and quadrants 1, 2, 4, 6, 8 and 9 are the overlapped genes showing the same expression in the two groups. The upper quadrant and right quadrant represent the gene counts in different expression intervals. **B** Biological processes (BPs) in which MDK-related, GBM-specific genes were significantly involved, with a focus on processes of interest in GBM. **C** KEGG pathway in which MDK-related, GBM-specific genes were significantly involved, with a focus on pathways of interest in GBM. **D** The enrichment score for the PI3K-Akt signaling pathway was significantly higher in the MDK-high GBM samples than in the MDK-low samples. MDK: midkine; GBM: glioblastoma; GSVA: gene set variation analysis; BP: biological process; KEGG: Kyoto Encyclopedia of Genes and Genomes

A total of 5537 MDK-related or 8556 GBM-specific DEGs were identified, of which 4841 overlapped between the two sets, which were common up and common down regulated in two group, and was defined as MDK-related, GBM-specific genes (Fig. 2A), we did not predefine a log (fold change) threshold in order to detect a maximum number of MDK-related, GBM-specific genes. GO and KEGG enrichment analysis of these MDK-related, GBM-specific DEGs showed involvement in “positive regulation of neuron differentiation”, “synapse organization”, and “cell morphogenesis” (Fig. 2B). The MDK-related, GBM-specific genes were also positively associated with functions including “cell cycle”, “apoptosis”, “PI3K-Akt signaling”, and “VEGF signaling” (Fig. 2C). Since multiple previous studies [40–42] have reported that the PI3K-Akt signaling pathway is associated with GBM, PI3K-Akt was subjected to GSVA. The PI3K-Akt signaling pathway enrichment score was significantly higher in MDK-high GBM samples than in MDK-low GBM samples (Fig. 2D), indicating that high MDK expression correlated with PI3K-Akt activation.

### An MDK-related transcriptional regulatory network in GBM

**Fig. 3:**
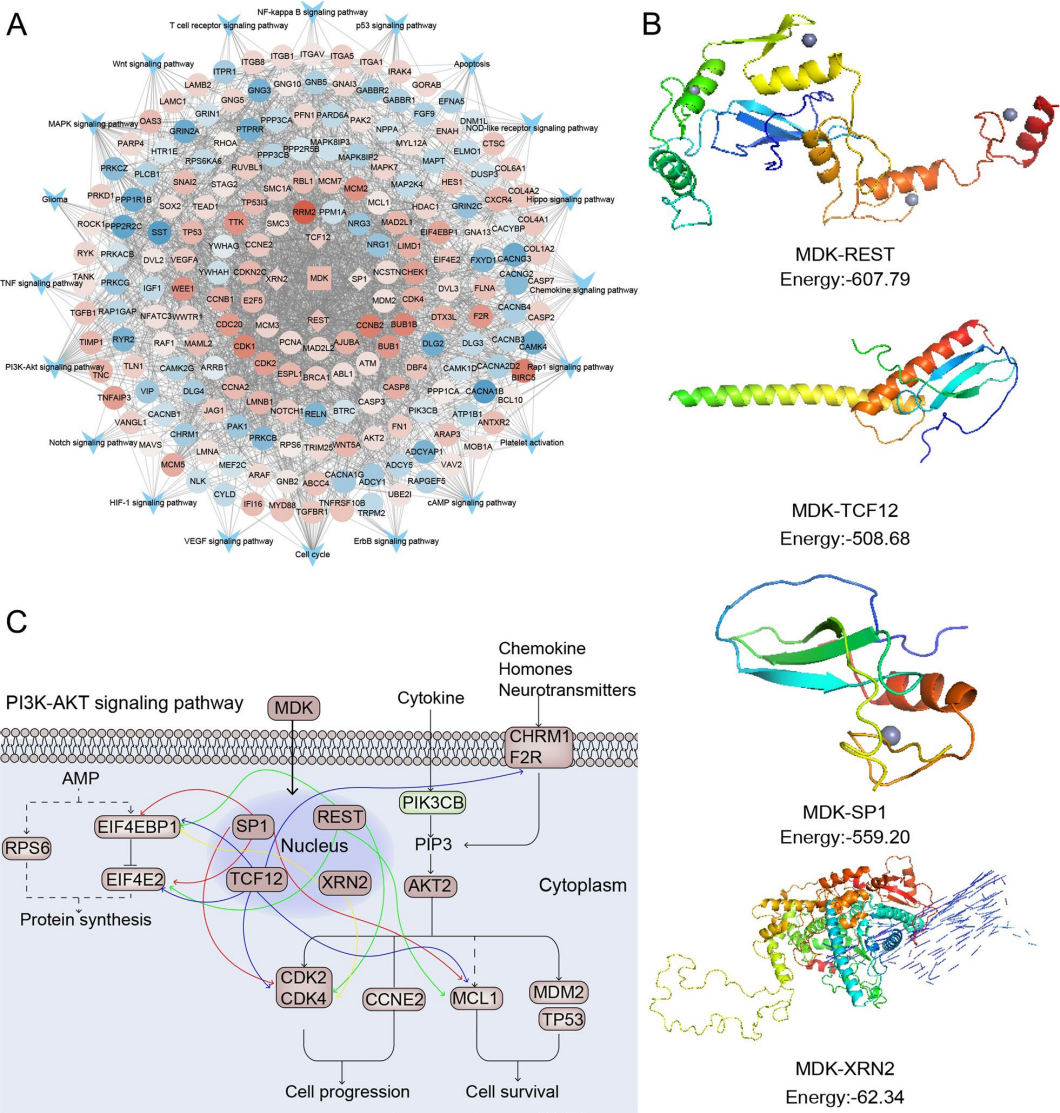
MDK-related regulation network in GBM. **A** The regulatory network of GBM development is regulated by MDK via TFs. The circle indicates pathway genes; the V shape, the signaling pathway; diamonds, TFs; and the square, MDK. **B** Molecular of MDK with four TFs: REST, TCF12, SP1 and XRN2. Docking energies (kcal/mol) are indicated below each complex; negative energies indicate that they may have a relationship. **C** MDK may regulate PI3K-Akt signaling by targeting TFs in GBM. The genes in this mechanism are the MDK-related, GBM-specific genes that are involved in the PI3K-Akt signaling pathway. The mechanism was adapted from the KEGG pathview, and lines are colored arbitrarily. MDK: midkine; GBM: glioblastoma; TF: transcription factor; KEGG: Kyoto Encyclopedia of Genes and Genomes

A comprehensive landscape of the regulatory pathway of MDK was constructed, which included 201 genes and 20 pathways (Fig. 3A), revealing a regulatory network through which MDK may participate in GBM development. This analysis highlighted interactions with four transcription factors (TFs) upstream of PI3K-Akt signaling: RE1 silencing transcription factor (REST), transcription factor 12 (TCF12), Sp1 transcription factor (SP1), and 5'-3' exoribonuclease 2 (XRN2). We found that MDK may have a relationship with these four TF (Fig. 3B). While in the result of CO-IP, we found that there is no significant combination between MDK and XRN2, TCF12, SP1 (Additional file 1: Figure S1). Our hypothesized mechanism of MDK regulation of GBM cell progression and survival via PI3K-Akt is summarized in Fig. 3C.

### MDK promotes the proliferation of GBM cells

**Fig. 4:**
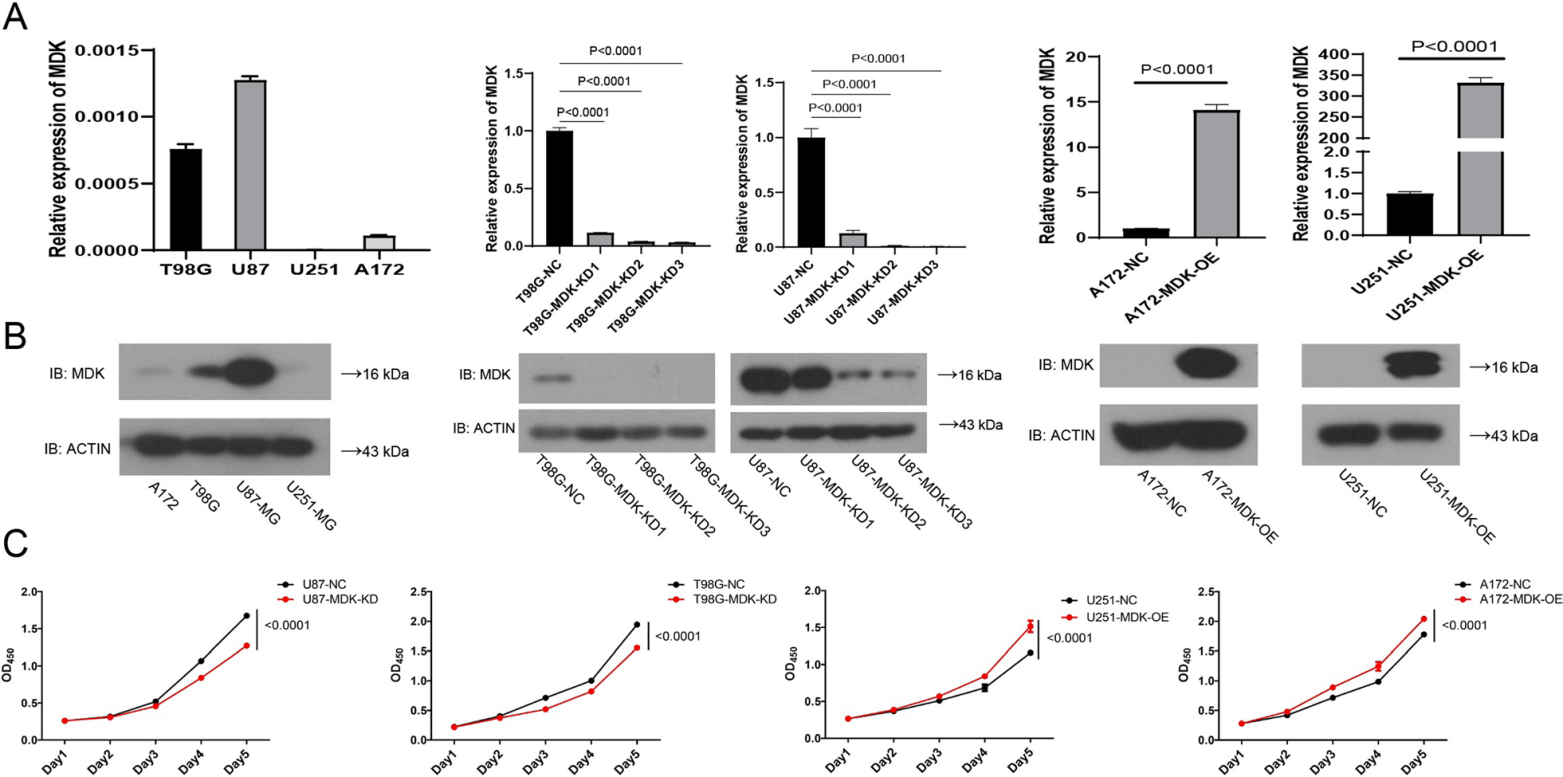
MDK promotes the proliferation of GBM cells. **A**, **B** Baseline MDK expression based on western blot and quantification, showing MDK levels in GBM cell lines in which MDK was knocked down (MDK-KD) or overexpressed (MDK-OE), and in negative control (NC) GBM cell lines. Beta-actin served as a loading control. **C** The proliferation of MDK-KD, MDK-OE, and NC GBM cell lines was analyzed using the CCK8 assay. MDK: midkine; GBM: glioblastoma; IB: immunoblotting

**Fig. 5:**
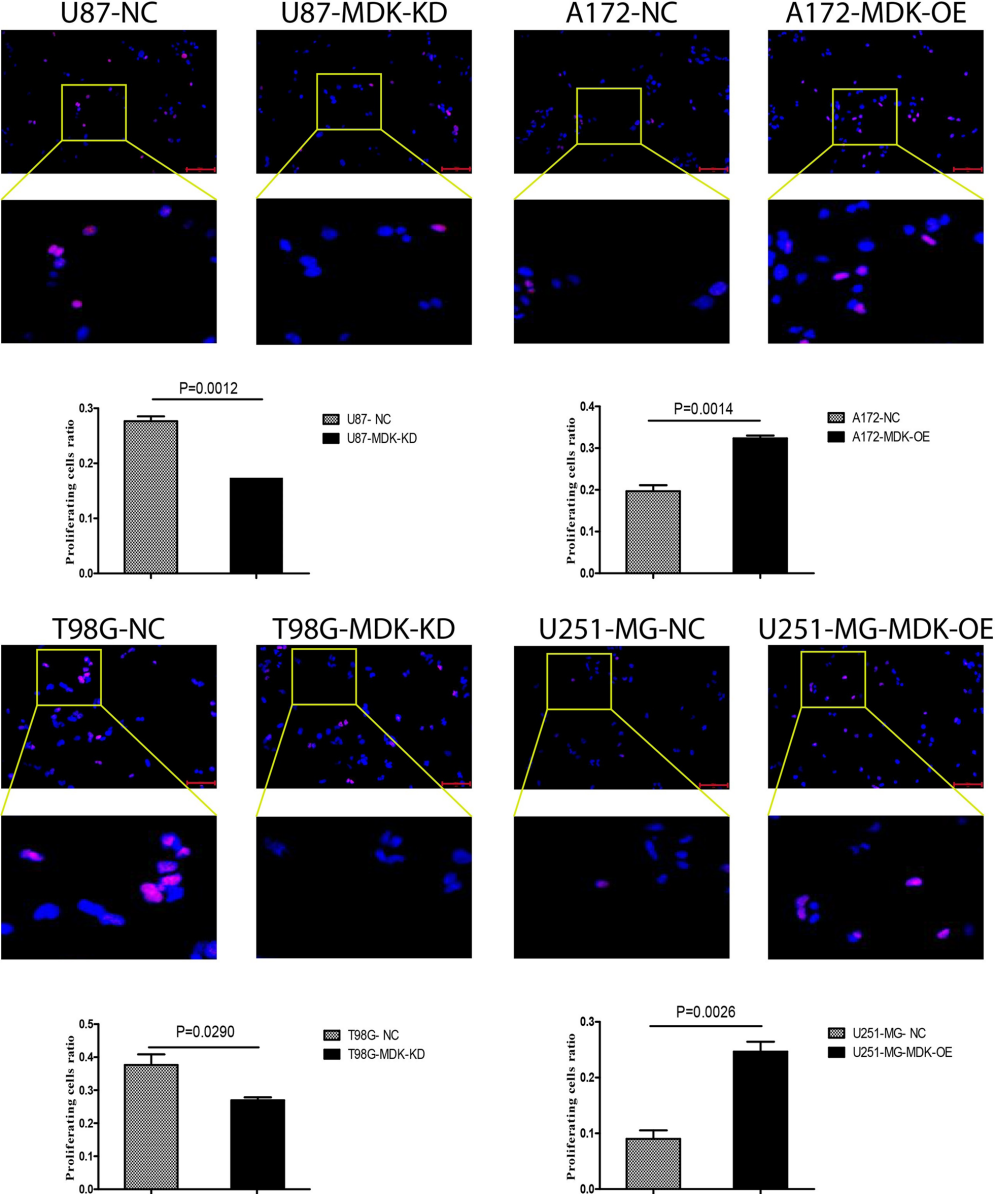
Cell proliferation was measured using the EdU assay in GBM cell lines overexpressing MDK (OE) or Knockdown MDK (KD) and in negative control (NC) lines. Representative fluorescence micrographs are shown. EdU: 5-ethynyl-2′-deoxyuridine; MDK: midkine

MDK showed high expression in human GBM cell lines T98G and U87-MG, and low expression in GBM cell lines A172 and U251-MG (Fig. 4A). We knocked down MDK in the high-expressing GBM cell lines to give rise to MDK-KD cells, and we overexpressed MDK in the low-expressing GBM cell lines to give rise to MDK-OE cells (Fig. 4B). The CCK8 assay showed that MDK-KD inhibited the proliferation of GBM cells compared with the negative control (NC), while MDK-OE increased proliferation (Fig. 4C). The same result was found in an EdU proliferation experiment (Fig. 5), confirming that MDK expression promotes GBM proliferation.

### Overexpression of MDK enhances the migration and invasion of GBM cells

**Fig. 6:**
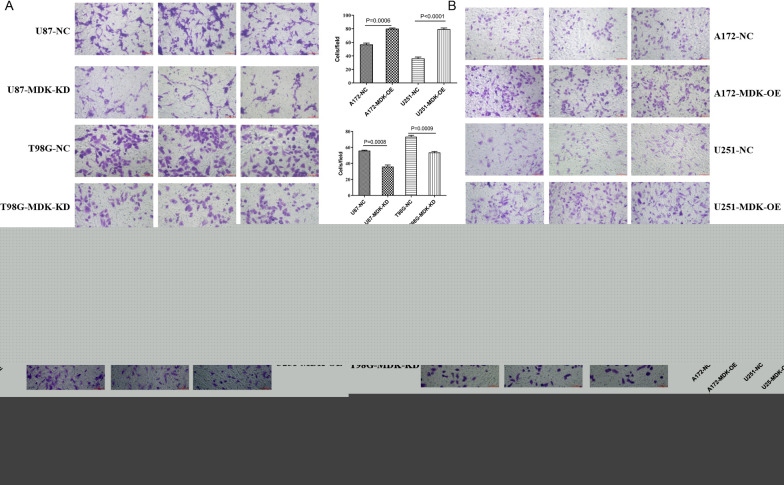
MDK promotes the migration and invasion of GBM cells. **A**, **B** The migration ability of GBM cell lines was assessed in the presence of MDK knockdown (MDK-KD) or overexpression (MDK-OE), or negative control (NC) conditions. Migration was measured in a Transwell assay. **C**, **D** The invasion ability of MDK-KD, MDK-OE, and NC GBM cell lines was measured using a Transwell assay with Matrigel. MDK: midkine; GBM: glioblastoma; NC: negative control

To understand the effect of MDK on GBM metastasis, the effects of MDK expression on GBM cell migration and invasion were assessed in vitro. Migration was measured with a transwell assay in each of the human GBM cell lines at baseline and after MDK knockdown or overexpression. Cell migration significantly decreased in the MDK-KD GBM cell lines (Fig. 6A) and increased in the MDK-OE lines (Fig. 6B) compared to baseline. The Matrigel-transwell invasion assay indicated that compared with the NC group, overexpression of MDK significantly enhanced the invasive ability of A172 and U251-MG GBM cells (Fig. 6D), while knocking down MDK inhibited invasion (Fig. 6C). Collectively, these results indicate that the upregulated MDK promotes GBM cell tumorigenesis and invasion.

### MDK activates PI3K-Akt signaling pathway in human GBM cells

We have clarified that, in line with our expectations, levels of p-Akt were significantly increased in the A172-MDK-OE cell line, but significantly decreased in the T98G-MDK-KD cell line. Contrary to our expectations, p-Akt levels were not increased in the U251-MDK-OE, nor were they decreased in the U87-MDK-KD cell line.

**Fig. 7:**
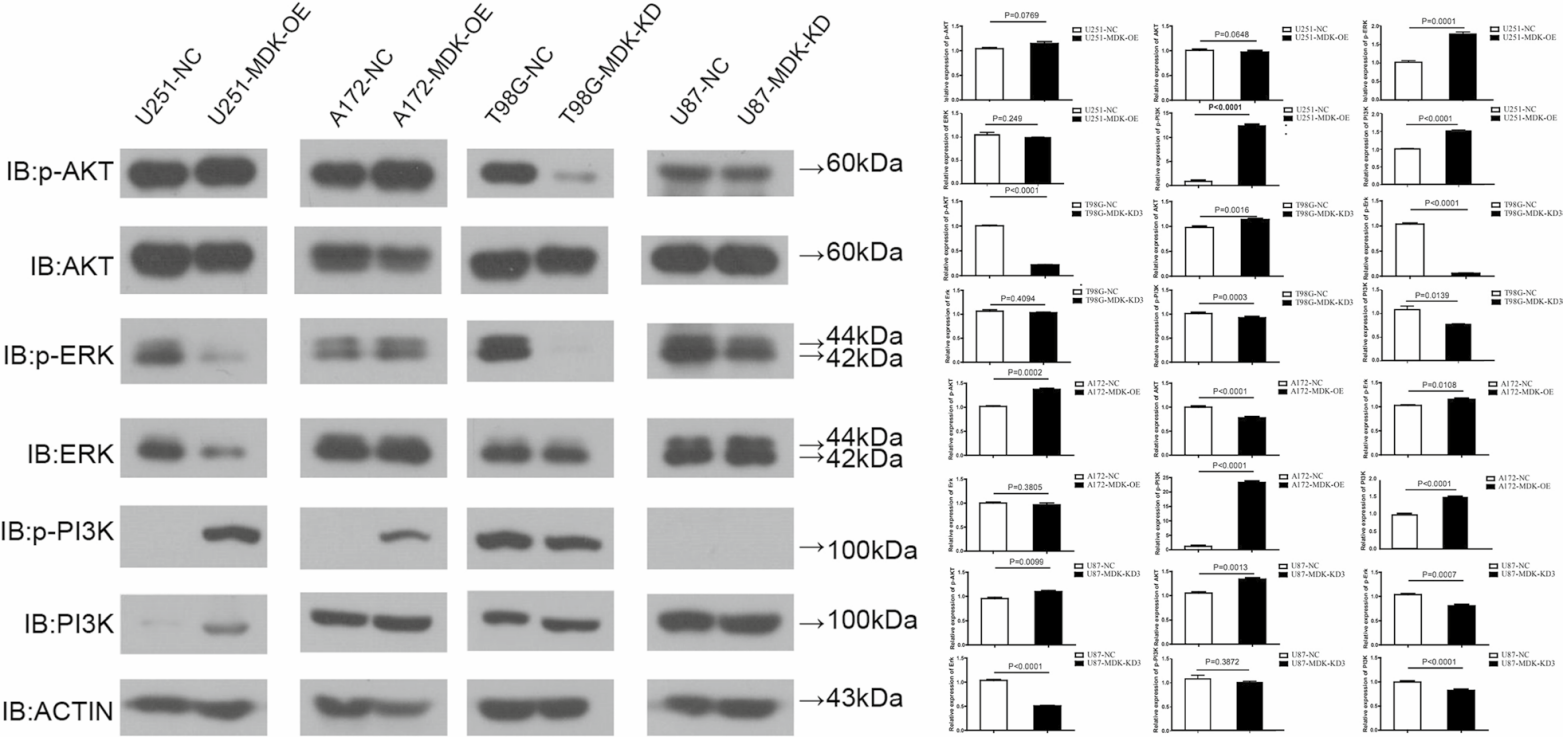
MDK acts via PI3K-Akt signaling to influence GBM cell proliferation, migration, and invasion. Western blot analysis of p-Akt, Akt, p-ERK, ERK, p-PI3K, PI3K and β-actin in GBM cell lines T98G and U87-MG in which MDK was knocked down (KD), and in GBM cell lines A172 and U251-MG in which MDK was overexpressed (OE). Negative control (NC) cells were also analyzed in parallel. MDK: midkine; GBM: glioblastoma; IB: immunoblotting

Consistent with our expectations, p-ERK levels decreased significantly in the MDK-KD cell line and increased significantly in the MDK-OE cell line. Also consistent with our hypotheses, p-PI3K was found only in U251-MDK-OE, T98G-MDK-KD and A172-MDK-OE cell lines; it was slightly but not significantly decreased in U87-MDK-KD (Fig. 7). We have emphasized that, taken together, these results suggest that the PI3K-Akt signaling pathway was activated in A172-MDK-OE cells and inhibited in T98G-MDK-KD cells.

## Discussion

Prior to the present study, we identified a relationship between MDK and poor GBM prognosis through bioinformatics [6], but the role of MDK in GBM remained unclear. Here we extend that work by validating the correlation in multiple datasets, characterizing the regulatory network of MDK in GBM, and honing in on a proposed mechanism of MDK action in GBM cell lines.

Growth factors play a key role in cell proliferation, differentiation, migration, and apoptosis, and they coordinate the growth signal response during development [43]. Many studies have found that the levels or function of cytokines are altered in cancer [44]. MDK has also been found to have abnormal expression in GBM [45]. In the present study, we demonstrated that the survival of GBM patients is inversely correlated with MDK expression, and that high MDK expression in GBM cell lines is associated with greater proliferation, migration, and invasion ability. To our knowledge, the present work is the first evidence that MDK may be a useful diagnostic and prognostic biomarker of GBM.

To investigate the effects of MDK on GBM progression, we extracted MDK-related, GBM-specific DEGs. Then, through functional enrichment analysis and correlation analysis, we found that MDK could activate the PI3K-Akt signaling axis by targeting four TFs: REST, TCF12, SP1 and XRN2. Many types of cancers involve aberrant activation of PI3K-Akt, which contributes to tumor cell proliferation, migration, and invasion [46, 47]. REST [48], TCF12 [49], and SP1 [50] are known to be master regulators that maintain GBM cell proliferation and migration. In contrast, there are few studies on the role of XRN2 in GBM. XRN2 has been shown to promote the EMT and subsequent metastasis of lung cancer [51]. Preliminary analysis of the present data further suggests that REST can regulate EIF4EBP1, EIF4E2, CDK4 and MCL1; TCF12 can regulate F2R, MCL1, CDK4, EIF4EBP1 and EIF4E2; SP1 can regulate EIF4EBP1, EIF4E2, CDK4, CDK2 and MCL1; and XRN2 can regulate CDK4 and EIF4EBP1. These potential interactions should be explored in further studies as mechanisms through which PI3K-Akt signaling is activated in GBM. Although the results of MDK binding to TCF12, SP1 and XRN2 in our experiments were not satisfactory, possibly due to too few validation samples. The combined experiment of MDK and REST was not implemented for technical reasons, so the CO-IP results of MDK and REST are not shown.

Consistent with a link between MDK and PI3K-Akt signaling, we conclude that the overexpression of MDK in human GBM cells probably relates to the activation of downstream signaling molecules through the PI3K-Akt pathway. Together, our data from patients, cell lines, and bioinformatics support the idea that MDK regulates the activity of certain TFs to promote PI3K-Akt signaling, which then increases GBM proliferation and invasiveness.

We conclude that MDK could function as an independent prognostic factor and a therapeutic target in the management of GBM. Though this study provides a solid theoretical basis for the treatment of GBM, the role of MDK in tumor progression in vivo is complex and needs further exploration. We have validated the importance of MDK in GBM progression in vivo*,* but our mechanistic studies were conducted largely in silico and in vitro.

There are limitations inherent to these experimental models, so our results should be verified in vivo. Our study was limited to preliminary exploration of potential molecular interactions. Future research will include the establishment of knockout mouse models to verify whether MDK activates PI3K-Akt signaling in the development of GBM.

## Supplementary Information

**Additional file 1: Figure S1.** The COIP of MDK and its target. COIP of MDK and its target. MDK was transfected into A172 cells respectively. Immunoprecipitation was performed by XRN2, TCF12 and SP1 antibody and immunoblotting. Co-IP, Co-immunoprecipitation; MDK, midkine; GBM, glioblastoma; IP, Immunoprecipitation; IB, immunoblotting; IgG, Immunoglobulin.

## Data Availability

The datasets generated and/or analyzed during the current study are available in the GEO database repository (https://www.ncbi.nlm.nih.gov/geo/query/acc.cgi?acc=GSE50161), and are available from the corresponding author on reasonable request.
