# Supplementary figures and images for: Midkine promotes glioblastoma progression via PI3K-Akt signaling
Source: Cancer Cell Int. 2021 Sep 23;21:509. doi: 10.1186/s12935-021-02212-3 (PMC8461913; doi:10.1186/s12935-021-02212-3)

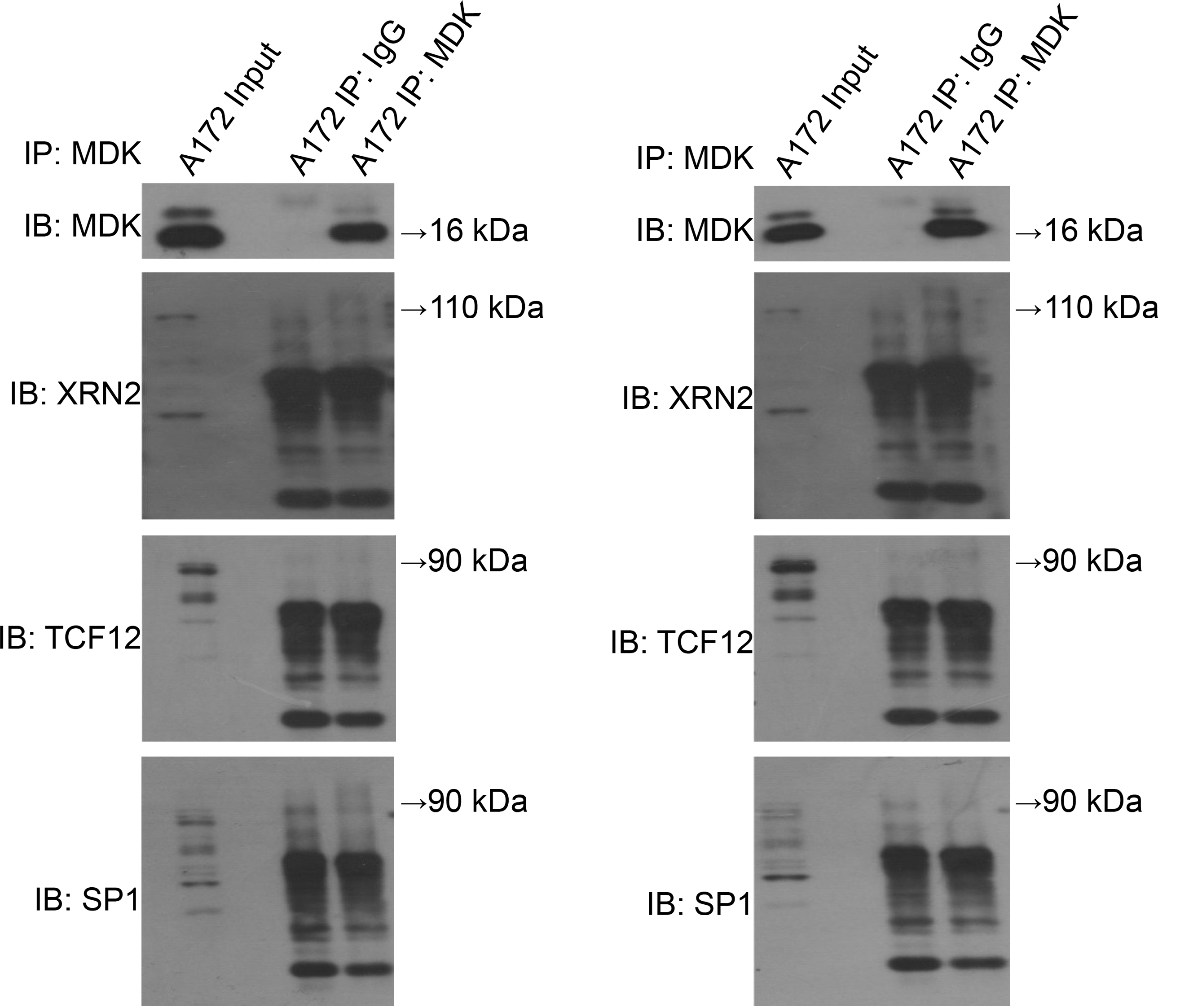

Supplement: Supplementary file 1 — Additional file 1: Figure S1. The COIP of MDK and its target. COIP of MDK and its target. MDK was transfected into A172 cells respectively. Immunoprecipitation was performed by XRN2, TCF12 and SP1 antibody and immunoblotting. Co-IP, Co-immunoprecipitation; MDK, midkine; GBM, glioblastoma; IP, Immunoprecipitation; IB, immunoblotting; IgG, Immunoglobulin. [file 12935_2021_2212_MOESM1_ESM.tif]
